# NLRP10 engages oxidized DNA through a Schiff-base mechanism and dissociates from NLRP3 upon inflammasome activation

**DOI:** 10.1038/s42003-025-09501-x

**Published:** 2026-01-22

**Authors:** Julia Elise Cabral, Angela Lackner, Wenjin Jiang, Sophia Lin, Haitian Zhou, Anna Wu, Courtney Demos, Minh Anh Pham, Reginald McNulty

**Affiliations:** 1 Laboratory of Macromolecular Structure, Department of Molecular Biology & Biochemistry, University of California, Irvine, CA USA; 2 Department of Pharmaceutical Sciences, University of California, Irvine, CA USA

**Keywords:** Immunology, Drug discovery

## Abstract

Mitochondrial DNA release into the cytosol is a critical event in innate immune activation, often acting as a damage-associated molecular pattern (DAMP) that triggers inflammasome assembly. Here, we demonstrate that NLRP3 is involved in the release of D-loop mtDNA into the cytosol. We further show that NLRP3 interacts with NLRP10. NLRP10-mediated oxidized DNA cleavage involves a Schiff base intermediate and is inhibited by small molecules known to inhibit glycosylases. These findings support a model where NLRP10 interaction with oxidized DNA may contribute to long-term senescence secretory phenotype and modulate inflammasome activation. Our study highlights a novel mechanism by which NLRP10 can respond to mitochondrial stress signals to influence innate immunity and suggests therapeutic potential for targeting these interactions in inflammatory diseases.

## Abstract (web summary)

The cytosolic innate immune sensor NLRP10 interacts with NLRP3 and mediates the cleavage of oxidized mitochondrial DNA

## Introduction

Mitochondrial DNA (mtDNA) serves as a potent damage-associated molecular pattern (DAMP) when released into the cytosol, triggering innate immune responses^1^. Oxidized mtDNA (ox-mtDNA) accumulates under conditions of cellular stress, mitochondrial dysfunction, and aging, linking it to the activation of key immune pathways, including the cGAS-STING and inflammasome axes^2–4^. Among these, the NLRP3 inflammasome plays a pivotal role in sensing mitochondrial distress and promoting IL-1β release^5,6^. Cryopyrin-associated periodic syndrome (CAPS) gain-of-function NLRP3 mutants have a higher affinity for oxidized DNA^7,8^. However, the mechanisms governing mtDNA oxidation, processing, cytosolic release, and the status of other NLR’s remain unclear and may be cell-type specific^9^.

NLRP3 has been shown to associate with ox-mtDNA^1^ and cells lacking NLRP3 exhibit reduced IL-1β secretion upon ox-mtDNA transfection^10^. NLRP3 directly binds ox-mtDNA with the pyrin domain preferentially binding the oxidized form^8^. NLRP3’s pyrin domain has glycosylase-like activity, allowing for the cleavage of oxidized DNA. Repurposed glycosylase inhibitors prevent both the interaction of NLRP3 with oxidized DNA and inflammasome activation^5^.

NLRP3 is known to associate with mitochondria^11^, and mitochondrial perturbation has been shown to release mtDNA fragments, including those from the D-loop region, via mPTP- and VDAC-dependent channels that can serve as activating signals for NLRP3^2^. This phenomenon implicates mtDNA in propagating inflammation and possibly reinforcing senescence-associated secretory phenotypes (SASP)^12^. While inflammasome activation is classically associated with caspase-1-mediated cytokine maturation, emerging evidence suggests that the persistence of ox-mtDNA in the cytosol may also contribute to prolonged inflammatory signaling and cellular senescence^13^.

NLRP10, an enigmatic member of the NOD-like receptor (NLR) family which lacks an LRR domain, has been implicated in immune modulation, but its precise function remains controversial. Initial reports indicate that NLRP10 primarily acts as a negative regulator of NLRP3-dependent inflammation rather than forming a classical inflammasome itself^14^. NLRP10 has also been linked to NF-κB regulation, a pathway controlled by transcription factor RelA and its inhibitor IκBα^15^. Reports suggest NLRP10 could affect priming by reducing^16^ or promoting NF-κB activity^17^. However, recent studies suggest that mitochondrial damage stimulates NLRP10 to interact with ASC, contributing to inflammasome formation and IL-1β secretion independent of NLRP3 in bone marrow-derived macrophages^18^. If NLRP3 and NLRP10 can associate and if their association is changed upon inflammasome activation is yet to be determined. Moreover, the role of ox-mt DNA relating to NLRP10 activation is questionable. NLRP10 shares conserved lysine and aspartic acid residues with hOGG1 base excision repair protein, similar to NLRP3, suggesting NLRP10 might be able to cleave ox-mtDNA^8^.

Herein, we present evidence that NLRP3 and NLRP10 can associate. Moreover, NLRP10 binds and cleaves oxidized DNA, suggesting an alternative role in DNA damage processing. Structural comparisons indicate that NLRP10 harbors a glycosylase-like fold typically associated with 8-oxoguanine DNA repair enzymes^19^ and shares conserved lysine and aspartic acid residues implicated in DNA cleavage. We hypothesize that NLRP10 functions as a novel oxidized DNA-processing enzyme that regulates inflammatory signaling and senescence by modulating cytosolic DNA levels.

## Results

### D-loop mitochondrial DNA release to the cytosol depends on an NLRP3-mediated pathway

**Fig. 1:**
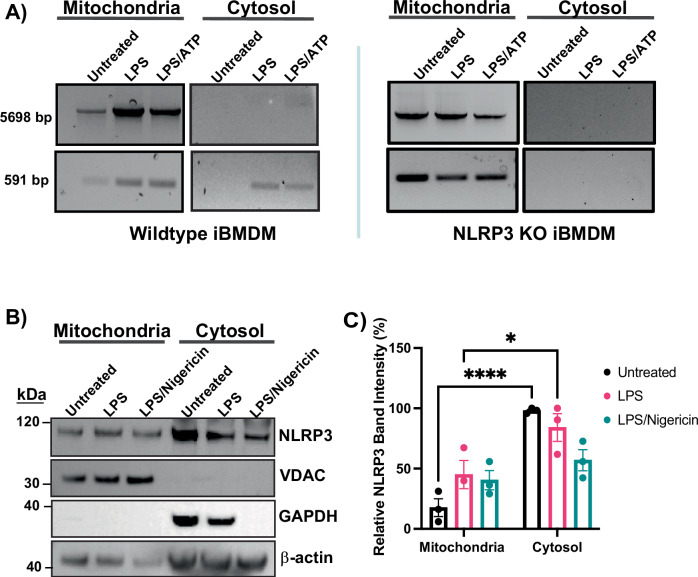
Mitochondrial DNA release is dependent on NLRP3. **A** Mitochondrial and cytosolic fractions were isolated from WT or NLRP3 KO iBMDMs treated with or without ATP. Amplification of 5698 and 591 bp fragments in both fractions are run on agarose gels. **B** Western blot analysis of mitochondrial and cytosolic fractions isolated from THP-1 cells, probed for NLRP3 on a PVDF membrane. β-actin serves as a loading control for the cytosolic fraction. VDAC confirms the absence of mitochondrial contamination in the cytosol, and GAPDH confirms the lack of cytosolic contamination in the mitochondria. **C** Quantification of NLRP3 in the mitochondria and cytosol with or without treatment with LPS and nigericin. Error bars: mean ± SEM, analyzed with two-way ANOVA. *N* = 3, ∗*p* = 0.0256, ∗∗∗∗*p* < 0.0001.

Drugs binding the NLRP3 pyrin domain have been shown to prevent NLRP3 from cleaving ox-mtDNA and inhibit both NLRP3 inflammasome activation and cytosolic mtDNA release^5^. This suggests NLRP3 might directly play a role in ox-mtDNA release. We performed PCR using primers for cleaved D-loop mtDNA in mitochondrial and cytosolic fractions of immortalized macrophages (iBMDMs)^2,20^ activated with ATP. PCR enabled detection of mtDNA large fragment, 5698 bp, in the mitochondrial fraction for both wildtype and NLRP3 knockout (KO) macrophages. Amplification of cleaved D-loop small fragment, 591 bp, was found in the mitochondria of both wildtype and NLRP3 KO iBMDM’s. However, cytosolic D-loop mtDNA was found in activated wild-type macrophages but was completely absent in activated NLRP3 KO macrophages (Fig. 1A and Supplementary Fig. 1). The lack of cleaved D-loop mtDNA in NLRP3-deficient macrophages indicates that NLRP3 is required for mtDNA cleavage and release to the cytosol.

### NLRP3 is predicted to associate with mitochondria via MTS-like N-terminal helix

NLRP3 inflammasome assembly is promoted by cardiolipin, a lipid found in the inner mitochondrial membrane. Upon stimulation, cardiolipin flips towards the outer membrane, serving as a nucleation site to initiate NLRP3 inflammasome assembly^21^. Since there are oftentimes variability in mouse compared to human cell immunology^22^, we checked if NLRP3 could associate with the mitochondria in human monocytes. We purified the mitochondrial and cytosolic fractions from monocytes stimulated with LPS and LPS/nigericin. To be sure our isolations were contamination-free, we checked for the presence of voltage-gated anion channel (VDAC) in the cytosolic and mitochondrial fractions. VDAC, which is localized specifically in the mitochondrial membrane, was only detected in the mitochondrial fraction and completely absent in the cytosol (Fig. 1B and Supplementary Fig. 2, 7A). We probed for GAPDH to ensure the mitochondria did not have cytosolic contamination. GAPDH has been reported to be involved in subcellular localization and to be secreted from cells following inflammasome activation^23^. We detected NLRP3 in both the mitochondrial and cytosolic fractions stimulated with either LPS or LPS/nigericin (Fig. 1C). The presence of NLRP3 in the mitochondrial fraction is consistent with previous findings^11^. Importantly, NLRP3 mitochondrial association has been shown to depend on the presence of mitochondrial antiviral protein (MAVS), a key adapter in RIG-I-like receptor pathways. Knockdown of MAVS reduces NLRP3 mitochondrial localization, supporting a connection between the two proteins^11^. AlphaFold structural predictions further suggest a close interaction between NLRP3 and MAVS, with NLRP3’s N-terminal helix positioned near the transmembrane domain of MAVS (Supplementary Fig. 3). Our data supports previous reports that NLRP3 interacts with the mitochondrial membrane through MAVS interaction and suggest NLRP3 association with the mitochondria is required for mt-oxDNA released to the cytosol.

**Fig. 2:**
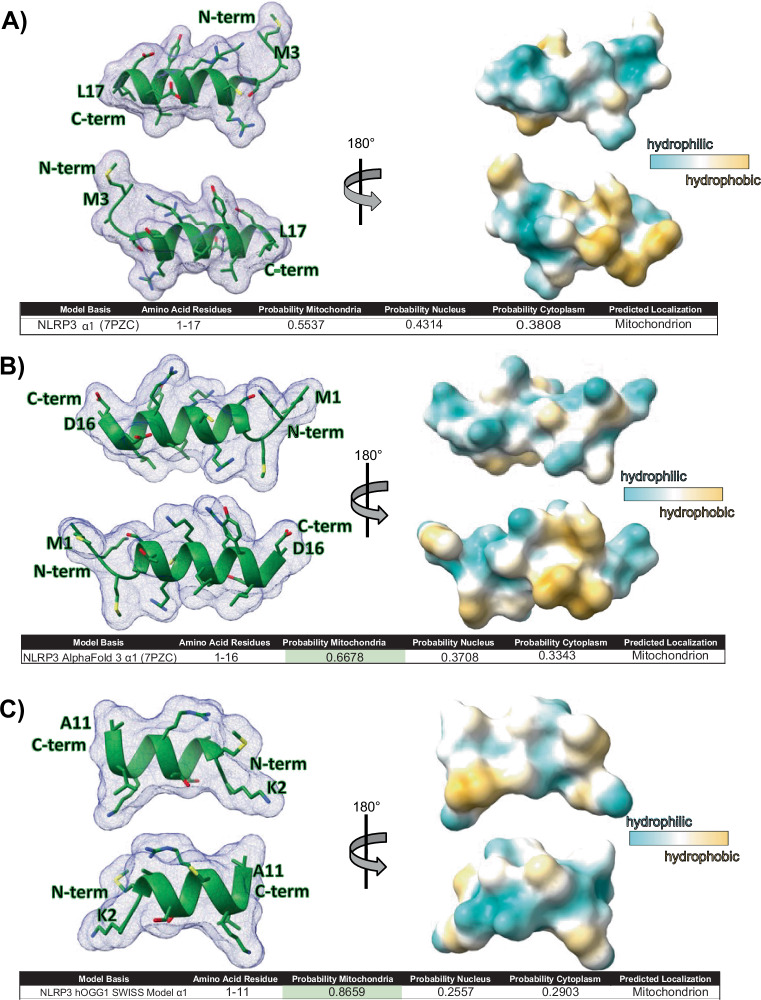
N-terminal NLRP3 helix is predicted to associate with the mitochondria. **A** NLRP3 helix one_3-17_ is amphipathic with hydrophobic areas in orange and hydrophilic areas in blue (PDB 7PZC). **B** NLRP3 pyrin domain AlphaFold3 model shows an amphipathic first helix_1-16_ with a predicted DeepLoc score of 0.6678. **C** NLRP3 SWISS-Model based on hOGG1 bound to oxidized DNA helix one_2-11_ shows regions of hydrophobicity and hydrophilicity. DeepLoc prediction software of N-terminal residues 1–11 predicts association with mitochondria with a score of 0.8659.

Although the fold of the death domain is shared amongst NLRP’s, they differ not only in percent identity, but the degree of structure/disorder at the N-terminus. Several software predictors of intrinsically disordered regions (IDR’s) identify the N-terminus of NLRP3 (and all other NLRP’s) as an IDR. This region could be the most subject to convert between order and disorder^8^. The N-terminal helix of NLRP3’s pyrin domain is required for both NLRP3 inflammasome activation and association with the mitochondria^11^. We investigated if this helix fits canonical descriptions of mitochondrial targeting signals (MTS), which are often characterized by an amphipathic N-terminal helix^24^. Using the program ChimeraX^25^, we found that NLRP3’s N-terminal helix has one side that is hydrophilic with polar and charged amino acids, while the opposite side was hydrophobic (non-polar) (Fig. 2). The inactive NLRP3 pyrin domain’s N-terminal helix spanned amino acids 3–17. The N-terminus through the first helix_1-17_ was not predicted by DeepLoc^26^ to associate with the mitochondria, with a mitochondria association probability of 0.5 (Fig. 2A). The N-terminus_1-16_ of the AlphaFold3 model of NLRP3 bound to oxidized DNA predicted a slightly higher mitochondrial association probability at 0.7 (Fig. 2B). However, analysis of NLRP3 bound to ox-mtDNA using SWISS-Model predicted the N-terminal helix_1-11_ had mitochondrial probability localization score that was much higher at 0.9 (Fig. 2C). This analysis is significant because it supports NLRP3 may make minor changes to support interaction with the mitochondrial membrane and ox-mtDNA. This data supports NLRP3’s N-terminal helix is structurally capable of interacting with the mitochondrial membrane, and supports N-terminal truncation studies showing the N-terminus is required to associate with MAVS^11^.

### IL-1β secretion is reduced by repurposed OGG1 inhibitors

**Fig. 3:**
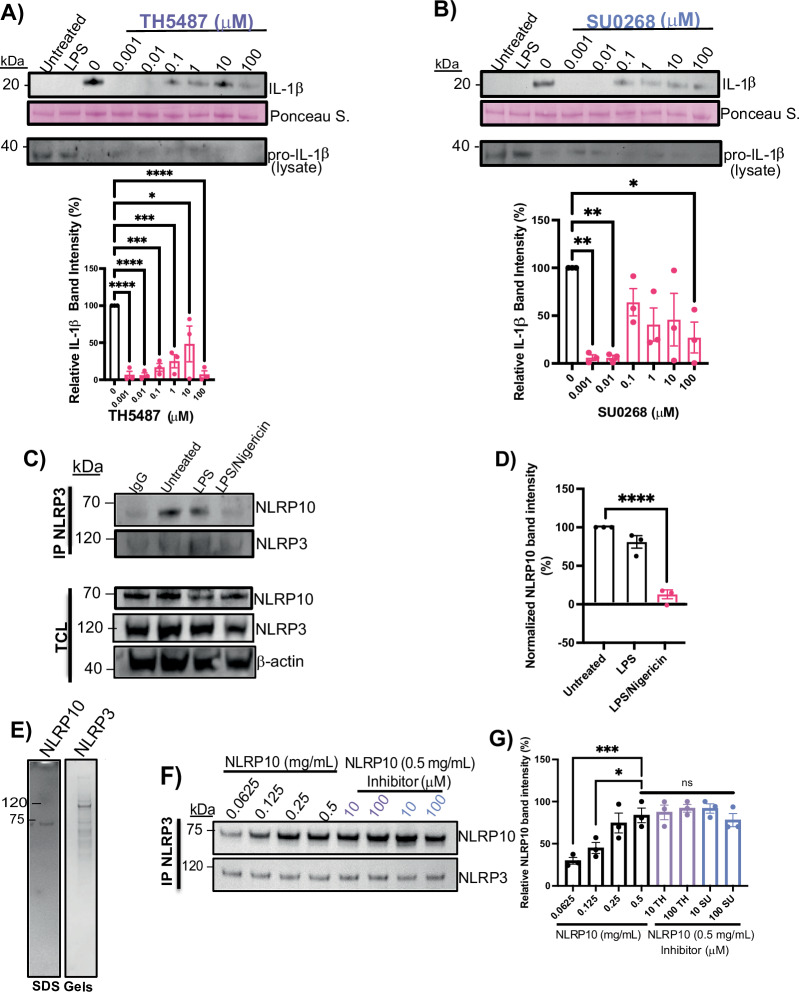
NLRP10 directly binds NLRP3, and the association decreases upon activation. **A** IL-1β western blots of LPS-primed and nigericin-activated THP-1 cells treated with increasing doses of TH5487. Ponceau S. stain serves as a loading control. Representative pro-IL-1β western blot. Quantification of IL-1β western blot data. Error bars: mean ± SEM, analyzed with one-way ANOVA. *N* = 3, ∗∗∗∗*p* < 0.0001, ∗∗∗*p* = 0.0003, 0.0008, ∗*p* = 0.0154. **B** IL-1β western blots of LPS-primed and nigericin-activated THP-1 cells treated with increasing doses of SU0268. Ponceau S. stain serves as a loading control. Representative pro-IL-1β western blot. Quantification of IL-1β western blot data. Error bars: mean ± SEM, analyzed with one-way ANOVA. *N* = 3, ∗∗*p* = 0.0026, ∗*p* = 0.0174. **C** NLRP3 IP pulldown of NLRP10 in LPS-primed nigericin-activated THP-1 cells. **D** Quantification of Western blot data. Error bars: mean ± SEM, analyzed with one-way ANOVA. *N* = 3, ∗∗∗∗*p* < 0.0001. **E** Coomassie SDS gels following affinity chromatography for NLRP10 (left) and size exclusion chromatography for NLRP3 (right). **F** NLRP3 IP pulldown of NLRP10 with purified protein. **G** Quantification of Western blot data. Mean ± SEM, one-way ANOVA. *N* = 3, ∗*p* = 0.0129, ∗∗∗*p* = 0.0007.

Targeting hOGG1’s base excision repair activity has been explored as a strategy to modulate inflammation. TH5487 and SU0268, inhibitors of OGG1, have also been reported to reduce NLRP3-mediated IL-1β secretion in iBMDM’s^5,27^. To test whether these small molecules inhibit IL-1β release in a human cell line that contains both NLRP3 and NLRP10, we first primed THP-1 cells with LPS, followed by increasing concentrations of either TH5487 or SU0268 prior to nigericin treatment. Western blot analysis revealed a decrease in IL-1β secretion with inhibition observed at the lowest tested concentration, 0.001 μM of both drugs (Fig. 3A, B and Supplementary Figs. 4A, 5A, 7B).

### Interaction between NLRP3 and NLRP10 is direct and is preserved in the unstimulated state

During NLRP3 activation, ox-mtDNA exits the mitochondria and activates the NLRP3 inflammasome, leading to an ASC speck in the cytosol. The presence of other pyrin domain-containing proteins could be antagonistic if they are present at the same time. This has been seen for pyrin-only protein 1 (POP1), which inhibits NLRP3 inflammasome activation^28^. Additionally, NLRP3 has been shown to co-localize with NLRP11 at ASC specks, where interaction between the two licenses inflammasome activation^29^. To test whether NLRP10 interacts with NLRP3, we performed co-immunoprecipitation using protein G-coated magnetic beads and a polyclonal NLRP3 antibody. We observed a stronger association between NLRP3 and NLRP10 in unstimulated THP-1 human monocytes than with inflammasome activation (Fig. 3C, D and Supplementary Figs. 4B, 5B, 7C). Treatment with MCC950 had no effect on the association between NLRP3 and NLRP10 in LPS/nigericin-treated cells compared to untreated cells, when examined by ELISA (Supplementary Fig. 4C).

To test whether NLRP10 interacts directly with NLRP3, we expressed full-length NLRP10 and NLRP3 in Expi293 cells and purified using affinity and size exclusion chromatography (Fig. 3E and Supplementary Fig. 5C). We then performed a co-immunoprecipitation using constant amounts of purified NLRP3 bound to the protein G-coated beads and incubated with increasing concentrations of NLRP10. We observed an increase in the amount of NLRP10 co-immunoprecipitating with NLRP3, and this interaction was not affected by the presence of SU0268 or TH5487 (Fig. 3F, G and Supplementary Figs. 5D, 7D). These results are significant because they suggest that the interaction between NLRP3 and ox-mtDNA may be modulated by the presence of NLRP10.

### NLRP10 has a glycosylase-like fold

**Fig. 4:**
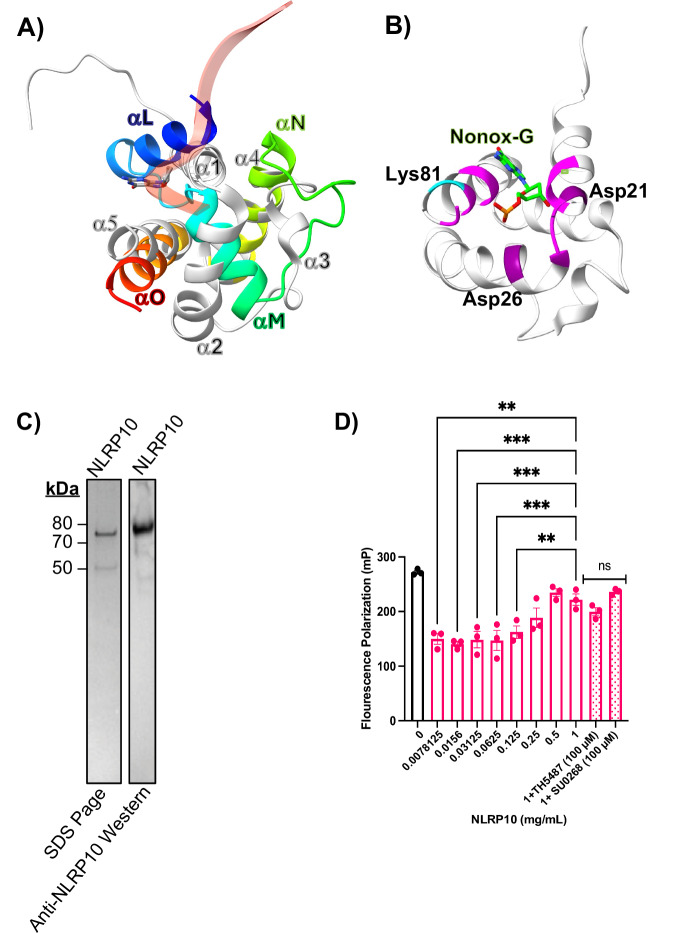
NLRP10 binds non-oxidized mitochondrial DNA. **A** Superposition of NLRP10 (gray PDB 2M5V) with hOGG1 (rainbow PDB 8VX6) interrogating a non-oxidized guanine. **B** Superposition from (**A**) with residues within 5 Å from non-oxidized guanine shown in magenta and lysine close to the catalytic site shown in cyan. **C** SDS Page gel following flag column purification of NLRP10 (left). PVDF membrane probed for NLRP10 following FLAG column purification (right). **D** Fluorescence polarization of cy5-labeled 20 bp non-oxidized DNA incubated with increasing concentrations of NLRP10. Error bars: mean ± SEM, analyzed with one-way ANOVA. *N* = 3, ∗∗*p* = 0.0011 and 0.0072 and ∗∗∗*p* = 0.0003, 0.0009, and 0.0007.

NLRP3 is able to bind both non-oxidized DNA and oxidized DNA similar to OGG1. Given the similarity of protein fold for NLRP3 and NLRP10 in the pyrin death domain fold, along with the identification of conserved lysine and aspartic acids critical for nucleophilic attack, we wanted to address if NLRP10 could bind non-oxidized DNA. Recent structures of hOGG1 bound to non-oxidized DNA illustrate an interrogation complex where non-oxidized guanine is flipped into the active site, but uncleaved. Superposition of NLRP10’s pyrin domain (1–102) with hOGG1 (248–325) bound to non-oxidized DNA^30^ illustrated a similar protein fold (Fig. 4A). NLRP10 H1 and hOGG1 helix αL are roughly the same length at 14.7 and 18.7 Å, respectively. These helices are not aligned. However, NLRP10’s H1 traverses the same direction and length of a disordered stretch of hOGG1. NLRP10 has a tight turn between Leu22 and Glu23. NLRP10 H2 traverses the same direction as hOGG1. NLRP10 H2 has four turns with a length of 21.1 Å, while hOGG1 αM has three turns and a length of 15.3 Å. Those helices are followed by a loop that turns in the same direction. NLRP10’s H3 is a 1-turn helix in the middle of the loop. The fold matches up again at NLRP10 H4 and hOGG1 αN.

hOGG1’s αN is 22.4 Å and has an extra turn, compared to NLRP10’s H4 which has a length of 19.2 Å. There is a tight turn for both proteins, followed by similar lengths of NLRP10 H5 and hOGG1 αO, which both have lengths of 22 Å. Those helices are traversing the same direction and just out of phase relative to each other. This suggests that NLRP10’s pyrin domain shares a similar fold with hOGG1. This raises the question as to if NLRP10 can interact with mitochondrial DNA in a method similar to hOGG1 and NLRP3.

### NLRP10 binds DNA

A cryo-EM structure of OGG1 bound to nucleosome non-oxidized DNA illustrates a non-oxidized guanine flipped out from DNA and interacting with the OGG1 active site^30^. Superposition of NLRP10 with OGG1 bound to a flipped non-oxidized guanine illustrates a similar interrogation complex. The putative active site has Asp21, Asp26, and Lys81 that could potentially participate in nucleophilic attack and excise the guanine if it were oxidized (Fig. 4B).

Repurposed OGG1 inhibitors that prevent NLRP3 from binding oxidized DNA have no effect on NLRP3 binding non-oxidized DNA^30^. So, we checked if NLRP10 could bind non-oxidized DNA and if the binding could be inhibited by repurposed inhibitors TH5487 and SU0268. We expressed and purified NLRP10 (75 kDa) via a FLAG affinity tag (Fig. 4C and Supplementary Fig. 6). We incubated 20 bp Cy5-labeled non-oxidized DNA with increasing concentrations of NLRP10. There was an initial decrease in fluorescence polarization (FP) signal upon adding 0.008 mg/mL NLRP10. However, the FP signal gradually increased with increasing concentrations of NLRP10. From 0.008 mg/ml to 0.5 mg/mL, the FP signal increased 36.1%, indicating NLRP10 could bind non-oxidized DNA (Fig. 4D). Addition of 100 μM TH5487 or SU0268 had no effect on binding non-oxidized DNA (Fig. 4D).

### NLRP10 forms a Schiff base and cleaves oxidized DNA

**Fig. 5:**
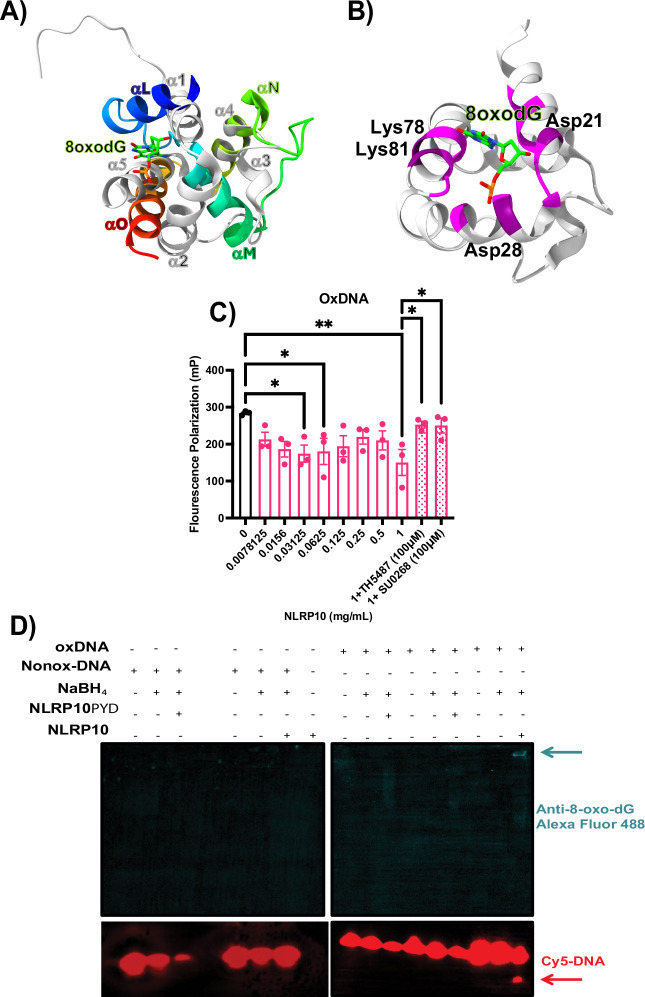
NLRP10 cleaves oxidized DNA. **A** Superposition of NLRP10 (gray PDB 2M5V) with hOGG1 bound to 8-oxo-dG (rainbow PDB 1EBM). **B** Superposition from (**A**), highlighting residues within 5 Å of oxidized guanine in magenta. Potential catalytic residues near the oxidized guanine are also indicated. **C** Fluorescence polarization of Cy5-labeled 20 bp oxidized DNA incubated with ATPγS and increasing concentrations of NLRP10, with or without TH5487 or SU0268. Error bars: mean ± SEM, analyzed with one-way ANOVA. *N* = 3, ∗*p* = 0.0256, 0.0379, 0.0412, and 0.0478, ∗∗*p* = 0.005. **D** Cy5-labeled 8-oxo-dG DNA incubated with either NLRP10_PYD_ or NLRP10 full length and sodium borohydride. Cy5-labeled DNA (red) anti-8-oxo-dG (teal). Cleaved ox-DNA (red arrow). Covalent complex of NLRP10 bound to 8-oxo-dG (teal arrow).

Given that NLRP10 could bind non-oxidized DNA and has a glycosylase-like fold (Fig. 4A), we examined if NLRP10 could interact with oxidized DNA and potentially cleave it. We first performed the superposition of hOGG1 bound to oxidized guanine-containing DNA. The superposition had an RMSD of 0.895 Å (Fig. 5A). The general path and alignment of the domain fold resembled that already shown in non-oxidized DNA. Residues potentially playing a role in catalysis within 5 Å of 8-oxo-dG included Asp26, Asp21, Lys78, and Lys81(Fig. 5B).

Since NLRP3 has glycosylase-like activity, and the fold is similar between NLRP10 and hOGG1, we examined if NLRP10 could cleave oxidized DNA. We incubated Cy5-labeled oxidized DNA with increasing concentrations of NLRP10 to probe for enzymatic activity (Fig. 5C). The FP signal from DNA alone to 1 mg/ml NLRP10, decreased 47.1%, suggesting the DNA was being cleaved. FP measurements in the presence of TH5487 and SU0268 increased back to the baseline value. This illustrates the repurposed drugs prevent NLRP10 from cleaving oxidized DNA.

hOGG1 glycosylase activity utilizes a conserved lysine that can perform a nucleophilic attack on 8-oxoguanine^31^, which will nick DNA if it is double-stranded, but cut the DNA if it is single-stranded. Prior to cutting, the conserved lysine makes a Schiff base intermediate with the oxidized DNA. Structures of hOGG1 bound to oxidized DNA have been trapped by mutating K249Q or chemically by using sodium borohydride (NaBH_4_)^31^. We incubated either non-oxidized or oxidized DNA with full-length NLRP10 or only the pyrin domain of NLRP10 (NLRP10_PYD_) and checked for Schiff base formation and DNA cleavage. Non-oxidized DNA did not show any cleavage for the pyrin domain alone or full-length (Fig. 5D). Cleavage was obtained with full-length NLRP10, as evidenced by an extra band underneath the primary oxidized DNA band in the lane (Fig. 5D). We investigated the mechanism of cleavage and found incubation of NLRP10 with sodium borohydride and oxidized DNA led to a Schiff base shifted high in the gel when probed with an antibody against 8-oxo-dG Alexa Fluor 488. This result was significant because we observed both Schiff base formation and oxidized DNA cleavage in the same lane (Fig. 5D).

## Discussion

We demonstrate that the release of mtDNA into the cytosol is dependent on NLRP3. The N-terminal helix of NLRP3 contains a predicted mitochondrial targeting sequence (MTS) and can associate with the mitochondrial membrane. To date, NLRP3 has not been found inside the mitochondria. But given that the inner mitochondrial membrane can become exposed to the cytosol under stress conditions, and NLRP3 has an amphipathic-like N-terminal helix, it is possible that NLRP3 might be responsible for the flipping of the inner membrane.

We further show that NLRP3 interacts with NLRP10, and that both proteins share a death domain with a structural fold akin to human glycosylase OGG1, displaying enzymatic activity specifically toward ox-mtDNA. To our knowledge, this is the first evidence that NLRP10 can form a Schiff base and has glycosylase-like activity to cleave oxidized mtDNA. Mechanistically, treating oxidized DNA and protein with sodium borohydride (NaBH_4_) facilitated trapping of the complex by reduction of the Schiff base. Meanwhile, a separate portion of the sample allowed simultaneous observation of NLRP10-mediated cleavage of oxidized DNA (Fig. 5D). These findings indicate that NLRP10 differentially recognizes oxidized versus non-oxidized DNA. This conclusion is further supported by the observation that OGG1 inhibitors TH5487 and SU0268 do not affect non-oxidized DNA but effectively inhibit enzymatic activity with oxidized mtDNA. Our data align with previous observations of NLRP3’s selective interaction with oxidized versus non-oxidized mtDNA^8^.

We illustrate herein that NLRP3 and NLRP10 directly associate with each other. Under inflammatory conditions, the association decreases in THP-1 cells, potentially supporting a mechanism by which NLRP10 binds NLRP3, preventing uncontrolled assembly of the NLRP3 inflammasome. NLRP10 activation has been linked to mitochondrial organelle damage^18^. Interestingly, ablation of mtDNA still triggers NLRP10 activation. The mitochondrial inner membrane cardiolipin is likely to participate in the NLRP10 activation just like it does for NLRP3^21^, potentially serving as a scaffolding platform. So, it is likely that either ligand is sufficient for NLRP10 activation. Furthermore, because NLRP10 and NLRP3 physically interact, their relative abundance and post-translational regulation may determine whether they act cooperatively or differently across cell types and stimuli.

Based on our findings that NLRP10 can cleave oxidized DNA similar to hOGG1, it is plausible that NLRP10 functions as a sensor of oxidative damage, particularly in mitochondrial dysfunction. Since mitochondrial oxidative stress and persistent DNA damage are hallmarks of cellular senescence^12^, NLRP10 may play an underappreciated role in linking inflammasome activation to senescence-associated inflammation. Oxidized DNA could act as a damage-associated molecular pattern (DAMP) that facilitates NLRP10 activation, even though it is not required for activation. Inflammasomes, including caspase-1-dependent signaling, have been implicated in the senescence-associated secretory phenotype (SASP)^32^, suggesting that NLRP10 may modulate the inflammatory microenvironment of senescent cells. Future studies should explore whether NLRP10 activation by oxidized DNA leads to persistent pro-inflammatory signaling and mitochondrial dysfunction, ultimately contributing to cellular aging and age-related pathologies, or whether NLRP10 cleavage of ox-mtDNA helps dampen excessive immune activation.

Both IL-1β and ox-mtDNA are considered SASP factors, and their relative predominance depends on the tissue type and cellular environment. Macrophages display an IL-1β dominant SASP with little ox-mtDNA-STING signaling^33^, whereas epithelial cells show the opposite profile^34^. Dermal keratinocytes have high IL-1β SASP in response to UV, while also having high ox-mtDNA cGAS/STING SASP in response to photoaging^35^.

More broadly, oxidized DNA from damaged mitochondria or nuclear sources engages innate immune receptors, including NLRP3 and potentially NLRP10, to activate inflammasomes and cGAS-STING, producing type I interferons (IFNs), IL-1β, and IL-18, which are key components of SASP. This sustained inflammatory response reinforces senescence by inducing paracrine signaling, where neighboring cells are driven into a senescent-like state, thereby amplifying tissue dysfunction. Additionally, oxidative stress-induced DNA damage leads to persistent activation of the DNA damage response (DDR), maintaining the senescent phenotype and preventing cell proliferation. Failure to clear oxidized DNA or senescent cells creates a chronic inflammatory microenvironment, accelerating aging and promoting age-related diseases, including fibrosis, neurodegeneration, and cancer. Given the connection between inflammasome activation initiated by pathogens^36^, diet^37^, and environmental toxicants, and oxidized DNA^38^, future work will involve elucidating the molecular determinants of recognition for oxidized DNA and repurposed small molecules using cryo-EM^39–41^.

## Methods

### Immortalized bone marrow-derived macrophage cell culture

Immortalized wild-type and knockout immortalized mouse macrophages^20^ were provided by Michael Karin at the University of California, San Diego. Cell vials were thawed in a water bath at 37 °C and resuspended in prewarmed media (DMEM (Thermo Fisher) supplemented with 10% heat-inactivated FBS (sigma) and 1% penicillin-streptomycin (Thermo Fisher)). Cells were spun at 300×*g* for 5 min at 4 °C to pellet the cells, and the media and cryoprotectant were aspirated. The cells were then resuspended in 5 mL of media, and the cell viability and concentration were determined by diluting cells 1:1 (10 μL of cells with 10 μL of Trypan Blue Stain) (Thermo Fisher). The dilution was then added to a Countess Cell Counting Chamber Slide (Thermo Fisher) and evaluated using a Countess 3 FL Automated Cell Counter (Thermo Fisher). Cells were then further diluted to a final concentration of 0.25 × 10^6^ cells/mL and plated onto 175 cm^2^ culture flasks (Corning) where they were grown at 37 °C and 5% humidity. Cells typically doubled within 24–48 h, after which they were scraped from the plate using Bio-One Cell Scrapers (Fisher Scientific), pelleted by centrifugation, counted, and expanded as previously explained.

### Immortalized bone marrow-derived macrophage NLRP3 activation

Cells with a viability greater than 95% were diluted to a concentration of 0.75 × 10⁶ cells/mL, ensuring they would reach 1 × 10⁶ cells/mL by the following day. Cells were plated in 6-well plates (Corning) with 2 mL of cells per well. Cells were incubated overnight at 37 °C and 5% humidity. The next day, 4 μL of 500x LPS (Thermo Fisher), final concentration 500 ng/mL, was added to each well and incubated for 4 h. After incubation, the LPS-only wells were harvested, and 4 mM ATP was added to each well for 1 h. Cells were scraped into 15 mL conicals and spun at 300×*g* for 5 min. The supernatant was removed, and the cell pellet was washed with PBS, and cell viability was checked. Cells were then spun at 500×*g*, and PBS was aspirated.

### Mitochondrial and cytosolic isolation in wild type/NLRP3 knockout iBMDM and THP-1s

Protocol is adapted from a previous study^2^. Briefly, following cell treatments, cell pellets were resuspended in 500 μL of cold mitochondrial extraction buffer (220 mM Mannitol, 70 mM sucrose, 20 mM HEPES-KOH, 1 mM EDTA, 2 mg/mL BSA, protease and phosphatase inhibitor cocktail tablet. Cells were passed through a 25-G syringe (BD Biosciences) 20 times on ice. The homogenized cell solution was then centrifuged at 1000×*g* for 15 min at 4 °C to pellet cell debris. The supernatant was then centrifuged at 12,000×*g* for 15 min to pellet the mitochondria. The supernatant was removed and labeled as the cytosolic fraction. The mitochondrial pellet was resuspended in 300 μL of cold RIPA buffer. Bradfords were performed on both fractions with the Quick Start Bradford Protein Assay Kit (Bio-Rad) prior to western blotting. NuPAGE 4–12% gels (Thermo) were run, and gels were transferred onto PVDF membranes. Membranes were blocked in 2.5% BSA and probed with pyrin targeting NLRP3 (Adipogen AG-20B-0014-C100), VDAC (Cell Signaling - #4661), and β-actin (Santa Cruz - #47778).

### Amplification of 591 base pair and 5698 base pair fragments from the cytosol and mitochondria of mouse iBMDMs

The mitochondrial and cytosolic fractions from immortalized bone marrow-derived macrophages were isolated as described above. The mitochondrial pellet was lysed using the lysis buffer from the Quick-DNA/RNA Miniprep Kit (Zymo Research), while the cytosolic fraction was used without further processing. DNA was then purified with the same kit, and the concentrations were measured using a NanoDrop spectrophotometer. PCR was performed to amplify both the large (5698 bp) and small (591 bp) fragments as previously described^2^. Briefly, PCR reactions were set up using the Phusion Hot Start 2x Master Mix (NEB #M0536) per the manufacturer’s protocol. For 5698 bp PCRs on the cytosolic fractions, 50 ng of DNA was used as the template. For all other reactions, 5 ng was used as the template. The following PCR conditions were used: hot start at 98 °C for 3 min, melting temperature of 98 °C for 10 s, annealing temperature of 60 °C, and extension temperature of 72 °C for 30 s (591 bp) or 3 min (5698 bp), followed by 72 °C for 20 min and kept at 4 °C. The PCR products were then analyzed on 1% agarose gels stained with ethidium bromide.

### Fluorescence polarization

A 100 μM stock of Cy5-labeled 20 base pair DNA (oxidized or non-oxidized (IDT)) was diluted 1:100 in DEPC-treated water to 1 μM. Protein was serially diluted (7–8 steps) with binding buffer (50 mM Tris HCl (pH 7.4), 100 mM NaCl, 2 mM MgCl_2_, 12% glycerol) starting at 1 mg/mL. ATPγS was included at a final concentration of 1 mM. For inhibitor conditions (TH5487 and SU0268), protein was preincubated with inhibitor and ATPγS for 1 h on ice prior to DNA addition. Samples without drugs were preincubated with ATPγS alone and received DNA at the same time point as the drug-treated samples. After 1 h, 10μ L of 1 mM DNA was added, and the mixtures were incubated on ice in the dark for 1 h. After incubation, samples were dispensed into a black-walled, clear-bottomed 384-well plate (Agilent #204623) with 30 μL per well. Fluorescence polarization was then read using a BioTek Synergy H1 plate reader with Generation 6 software (Agilent) at an excitation of 620/40 and an emission of 680/30, specific to Cy5. The polarization values were calculated based on the parallel and perpendicular light and G-factor-corrected.

### NLRP10 pyrin, NLRP10 full-length, and NLRP3 protein expression and purification

NLRP10 plasmid was a gift from Thomas Kufer (Addgene plasmid # 38141). For the NLRP10 pyrin domain, site-directed mutagenesis was performed (NEB #E0554S) to introduce a stop codon after the 100^th^ amino acid. All plasmids were sequenced, grown at large scale in DH5α cells, and then the DNA was purified with PureLink HiPure Plasmid Maxiprep kit (Thermo Fisher). The proteins were each expressed in 500 mL of media using the Expi293 Expression System (Thermo Fisher). Briefly, cells were grown to roughly 3 × 10^6^ cells per milliliter and ≥95% live cells. Enhancers were added 16 h after transfection.

Once the cells reached a viability of <80% live cells, they were harvested by spinning at 1200 rpm in a swinging bucket rotor (JS-4.750, Beckman). After centrifugation, the media and dead cells was aspirated, and the pellet was washed with cold PBS to remove residual media. The cells were then harvested again by centrifugation, and the PBS was aspirated. The protein was purified on the AKTA Avant. Cells were resuspended in lysis buffer (1 mm PMSF, 50 mM Tris HCl pH 7.4, 300 mM NaCl, 0.1% SDS, 10% glycerol, 1% Triton X-100, protease inhibitor tablets (Sigma)). The lysate was then sonicated for 42 s in intervals of 2 s on and 8 s off. The lysate was then clarified by spinning at 100,000×*g* for 1 h and subsequently passed through a 0.45-μm filter prior to affinity chromatography. For NLRP10 purification, the chromatography column (XK16) was packed with flag resin and equilibrated with binding buffer (150 mM NaCl, 50 mM Tris, 0.05% NP-40). The NLRP3 construct was loaded onto a HisTrap FF crude column equilibrated with (20 mM Tris, 200 mM NaCl, 10% glycerol, 1 mM DTT, pH 7.4). NLRP10 protein was eluted off with elution buffer (0.1 M glycine, 0.08% NP-40) into a 96-well 2 mL fraction collector containing Tris HCl at a final concentration of 60 mM. NLRP3 was eluted off in a four-step gradient (20 mM Tris, 200 mM NaCl, 10% glycerol, 1 mM DTT, 500 mM imidazole, pH 7.4). Fractions were analyzed on a total protein NuPAGE 4–12% Bis-Tris gel run at 200 V for 30 min. Fractions were further analyzed with PVDF membrane western blots blocked in 2.5% BSA and probed with NLRP3 pyrin targeting (Adipogen) or an NLRP10 NACHT targeting antibody (Cell Signaling). Based on the results from the gels and westerns, similar NLRP10 fractions were pooled and concentrated in a 50 kD molecular weight cut-off spin concentrator (Sigma). NLRP10 was then dialyzed in 500 μL 10 kD dialysis bags (Sigma) for 4 h at 4 °C into dialysis buffer (20 mM Tris HCl, 200 mM NaCl, 0.08% NP-40, 10% glycerol, 1 mM DTT). Following affinity chromatography, NLRP3 was then loaded onto a HiLoad 16/600 Superose 6 pg size exclusion column and run in a buffer containing 20 mM Tris, 200 mM NaCl, 10% glycerol, 1 mM DTT, pH 7.4.

### ChimeraX and hydrophobicity maps

The inactive structure of NLRP3 (PDB 7PZC)^39^ was opened in ChimeraX. The N-terminus through the end of the first helix was selected (3–17). A separate .pdb file was saved to isolate this helix, and the hydrophobicity was displayed. To generate the AlphaFold model, the sequence of NLRP3’s pyrin domain (PDB 7PZC, amino acids 1–91) was inputted into AlphaFold3 using the Colab server. Next, the sequence of single-stranded oxidized DNA (PDB 1EBM, Chain C) was added to the same query. Alphafold generated five putative models, and the model with the highest combined confidence score was evaluated using ChimeraX. The N-terminus through the end of the first helix was selected (1–16), and the hydrophobicity displayed on the isolated helix. To generate the SWISS-Model, the amino acid sequence of hOGG1_(249–325)_ that aligned with the NLRP3 pyrin_(1–91)_ domain was saved as a.pdb file. The sequence was uploaded to SWISS-Model in their user template modeling input option as the template file. Then, NLRP3 amino acids 1–85 was loaded as the target file (amino acids 1–90 did not generate a model). The SWISS-Model projection produced one model of NLRP3 based on the template hOGG1 structure. The N-terminus through the end of the first helix (2–11) was isolated in ChimeraX, and the hydrophobicity was shown.

### AlphaFold modeling of MAVS and NLRP3

AlphaFold3 was used to generate structural predictions of 3 NLRP3 monomers (AAI43360.1) interacting with MAVS (NP_065797.2). The predicted structures were opened in ChimeraX. All structures were predicted to interact. The model selected highlighted the proximity of NLRP3’s N-terminal helix and the MAVS transmembrane domain.

### THP-1 cell culture

THP-1 cells were purchased from Invivogen. Cells were thawed in a water bath and resuspended in 5 mL of RPMI 1640 without phenol red (Thermo Fisher), supplemented with 1× non-essential amino acids (Thermo Fisher), 1× Penicillin-Streptomycin-Glutamine (Thermo Fisher), 1× sodium pyruvate (Thermo Fisher), and 10% heat-inactivated FBS (Thermo Fisher). The cells were centrifuged at 300×*g* for 5 min, and the cryoprotectant and media were aspirated. Cells were then resuspended in 5 mL of media, counted using a Countess 3 FL Automated Cell Counter (Thermo Fisher), and diluted to 0.25 × 10⁶ cells/mL. Cells were incubated at 37 °C and 5% humidity.

### THP-1 NLRP3 activation assay

THP-1 cells with a viability greater than 95% were diluted to a concentration of 0.75 × 10⁶ cells/mL the day before the assay was performed. Cells were plated in six-well plates (Corning) with 2 mL of cells per well. Once cells reached 1 × 10⁶ cells/mL, LPS (Thermo Fisher, 500X, 2.5 mg/mL stock) was added, and cells were incubated for 16 h at 37 °C, 5% CO₂. For co-immunoprecipitation and ELISA, cells were primed with LPS at 5 µg/mL (1:500; 1X) to ensure robust priming for protein–protein interaction capture. For inflammasome activation/IL-1β assays, LPS was used at 500 ng/mL (prepared with a 1:10 dilution). After 16 h, cells were treated with increasing concentrations of either TH5487 or SU0268 for 1 h. MCC950 was added following LPS incubation at 10 μM. Following the incubation, LPS wells were harvested, and nigericin was added for 1 h (20 µM). Cells were transferred to 15 mL conical tubes, spun at 300×*g*, and the supernatant was saved for western blot analysis. Cells were washed with cold PBS, and cell viability was determined as described above. Cells were then spun at 500×*g*, and PBS was aspirated.

### Co-immunoprecipitation with THP-1 cells

THP-1 cell pellets were lysed with gentle lysis buffer (50 mM Tris HCl, 150 mM NaCl, 5 mM EDTA, 1% NP-40, and 1 mM PMSF). Bradfords were run on cell lysates (Bio-Rad) to ensure equal loading onto the beads. PureProteome Protein G magnetic beads (Sigma) were resuspended, and 50 μL were removed for each replicate. Tubes containing beads were placed on a magnet, and the storage puffer was discarded. Beads were washed with 500 μL of wash buffer (PBS supplemented with 0.1% Tween). Beads were resuspended in wash buffer and incubated with polyclonal NLRP3 antibody (1:200, ABclonal A12694) or normal rabbit IgG (Cell Signaling #2729) overnight at 4 °C. Antibodies were added at the same final concentration of 11.25 μg/mL. Beads were placed on the magnet and washed with the wash buffer to remove unbound antibody. Equal amounts of protein were incubated with the beads overnight at 4 °C. The LPS/nigericin cell lysate was used for the control beads. Beads were pulled back and the unbound sample was removed. Beads were washed with the wash buffer three times. NuPAGE sample buffer and reducing agent (Thermo Fisher) were added at 1x and boiled for 10 min at 80 °C. Beads and samples were loaded together onto NuPAGE 4–12% gels (Thermo Fisher). Gels were transferred onto PVDF membranes, blocked with 2.5% BSA, and probed for NLRP3 (1:10,000 Adipogen AG-20B-0014-C100) and NLRP10 (1:1000 R&D systems MAB6606). Total cell lysate was probed for NLRP3 (1:10,000 Adipogen AG-20B-0014-C100), NLRP10 (1:1000 R&D systems MAB6606), and β-actin (Santa Cruz - #47778).

### Co-immunoprecipitation with purified NLRP3 and NLRP10

Full-length NLRP10 and full-length NLRP3 were purified as described above. Bradfords were run to assess protein concentration. For each replicate, 50 μL of PureProteome protein G beads (Sigma) were washed with 500 μL of wash buffer. Beads were resuspended in 100 μL of wash buffer and incubated with polyclonal NLRP3 antibody (1:200, ABclonal A12694) for 2 h at 4 °C. Following incubation, the tubes were placed on the magnet, and the beads were washed with 500 μL of wash buffer three times on ice. Replicates were divided, and purified NLRP3 was added at a final concentration of 0.5 mg/mL. Beads were incubated with protein for 1 h at 4 °C. During the incubation, NLRP10 was serially diluted, and the highest concentration was incubated with either 10 or 100 μM of either TH5487 or SU0268 for 1 h. After 1 h, the beads were placed on the magnet and the unbound protein was removed. Beads were washed with 500 μL of wash buffer three times on ice. NLRP10 was added to the beads and incubated. Following incubation, samples were placed on the magnet, and unbound NLRP10 was removed, and the beads were washed with wash buffer three times on ice. NuPAGE sample buffer and reducing agent (Thermo Fisher) were added at 1x and boiled for 10 min at 80 °C. Beads and samples were loaded together onto NuPAGE 4–12% gels (Thermo Fisher). Gels were transferred onto PVDF membranes, blocked with 2.5% BSA, and probed for NLRP3 (1:10,000 Adipogen AG-20B-0014-C100) and NLRP10 (1:1000 R&D systems MAB6606).

### ELISA to assess NLRP3-NLRP10 interaction

Half-area microtiter plate (Fisher Scientific 0720037) was coated with NLRP3 capture antibody (1:200 in PBS, ABclonal A12694) and incubated overnight at room temperature. The next day, the capture antibody was aspirated, and the wells were washed with wash buffer three times (0.05% Tween 20 in PBS, pH 7.2). The plate was blocked in 1% BSA in PBS for a minimum of 1 h at room temperature. The blocking solution was removed, and wells were washed following incubation. Bradfords (Bio-Rad) were performed on cell lysates and normalized prior to addition to the wells. Lysates were incubated for 2 h at room temperature prior to aspiration and washes. NLRP10 detection antibody was diluted 1:1000 in 2.5% BSA TBST and added to each well (R&D Systems MAB6606) for 2 h at room temperature. Solution was aspirated, wells washed, and mouse secondary antibody was diluted 1:1000 in 1% BSA PBS and incubated for 2 h (Cell Signaling #7076). Following incubation, the solution was removed, and wells were washed prior to the addition of TMB substrate solution (Thermo 34021) for 20 min. The stop solution was added and the optical density was read with a BioTek Synergy H1 plate reader with Generation 6 software (Agilent) set to 450 nm. The 450 absorbance read was corrected by subtracting the values at 540 nm.

### Schiff base trapping of NLRP10 and oxidized DNA

The protocol was adapted from a previous study. Briefly, full-length NLRP10 (0.85 mg/mL) or NLRP10_1–100_ (0.25 mg/mL) was added to a reaction buffer containing 50 mM NaBH_4_, 25 mM potassium phosphate (pH 6.8), 1 mM DTT, and 1 mM EDTA at their final concentrations. A 100 μM stock of Cy5-labeled 20 base pair DNA (oxidized (AATCTACCAi8oxodGTCCTCCCTCA3Cy5Sp) or non-oxidized (AATCTACCAGTCCTCCCTCA3Cy5Sp)(IDT)) was diluted 1:100 in DEPC-treated water to a final concentration of 1 μM. A volume of 5 μL DNA was added to each tube and incubated at 37 °C for 30 min, followed by placement on ice for 1 h. Readouts were performed immediately after incubation or several days later, following incubation at 4 °C. Samples were analyzed on 4–20% Tris-glycine gels (Thermo Fisher). Samples were mixed with orange loading dye (NEB) containing SDS, boiled at 70 °C for 10 min, and loaded onto the gel. Fluorescent signals were detected both after electrophoresis and following transfer onto a PVDF membrane. Oxidized DNA signals were further analyzed via western blot after UV crosslinking of the membrane for 5 min. Blots were blocked with 2.5% BSA at room temperature and probed with an 8-oxo-dG antibody (1:1000, Rockland). The secondary antibody, Alexa Fluor 488 goat anti-mouse IgG/IgM (H + L) (1:2500, Thermo Fisher), was incubated overnight at 4 °C, and membranes were imaged using iBright software.

### Normalization of supernatant samples

Bradford assays were performed to normalize protein concentrations in both supernatant and lysate samples. The Quick Start Bradford Protein Kit 2 (Bio-Rad) was used to generate a standard curve. Samples were then read in duplicate and averaged to determine protein concentration. To verify equal loading of supernatant samples, Ponceau stains (Thermo Fisher) were performed on PVDF membranes following chemiluminescence. Blots were stained and destained according to the manufacturer's directions. Lysate samples utilized Western blot of a housekeeping protein such as β-actin, GAPDH, or VDAC (for mitochondrial fractions).

### Statistics and reproducibility

A one-way or two-way ANOVA was used to conduct all statistical analyses herein. All statistical analyses were performed as indicated in the Figure legends, where *N* represents the number of replicates. The data all represent mean ± SEM, where *p* values <0.05 were considered statistically significant. Normalization was performed for quantified data to the control.

### Reporting summary

Further information on research design is available in the Nature Portfolio Reporting Summary linked to this article.

## Supplementary information

Supplementary Information

Reporting Summary

## Data Availability

Uncropped westerns and gels can be found in the Supplementary Information file. All numerical data used to generate the data shown are available at the Figshare repository: https://figshare.com/s/cb80e47bc3bd0159cf54. Additional data that support the findings of this study are available from the corresponding author upon reasonable request.

## References

[1] Shimada, K. et al. Oxidized mitochondrial DNA activates the NLRP3 inflammasome during apoptosis. *Immunity* **36**, 401–414 (2012). 22342844 10.1016/j.immuni.2012.01.009 PMC3312986

[2] Xian, H. et al. Oxidized DNA fragments exit mitochondria via mPTP- and VDAC-dependent channels to activate NLRP3 inflammasome and interferon signaling. *Immunity* **55**, 1370–1385.e1378 (2022). 35835107 10.1016/j.immuni.2022.06.007 PMC9378606

[3] Gluck, S. et al. Innate immune sensing of cytosolic chromatin fragments through cGAS promotes senescence. *Nat. Cell Biol.* **19**, 1061–1070 (2017). 28759028 10.1038/ncb3586 PMC5826565

[4] Liao, C. Y., Lei, C. Q. & Shu, H. B. PCBP1 modulates the innate immune response by facilitating the binding of cGAS to DNA. *Cell Mol. Immunol.* **18**, 2334–2343 (2021). 32415261 10.1038/s41423-020-0462-3 PMC8484664

[5] Lackner, A. et al. Small molecule inhibitor binds to NOD-like receptor family pyrin domain containing 3 and prevents inflammasome activation. *iScience* **27**, 110459 (2024). 39104412 10.1016/j.isci.2024.110459 PMC11298654

[6] Broderick, L. & Hoffman, H. M. IL-1 and autoinflammatory disease: biology, pathogenesis and therapeutic targeting. *Nat. Rev. Rheumatol.* **18**, 448–463 (2022). 35729334 10.1038/s41584-022-00797-1 PMC9210802

[7] Hoffman, H. M., Mueller, J. L., Broide, D. H., Wanderer, A. A. & Kolodner, R. D. Mutation of a new gene encoding a putative pyrin-like protein causes familial cold autoinflammatory syndrome and Muckle-Wells syndrome. *Nat. Genet.* **29**, 301–305 (2001). 11687797 10.1038/ng756 PMC4322000

[8] Cabral, A. et al. Differential Binding of NLRP3 to non-oxidized and Ox-mtDNA mediates NLRP3 inflammasome activation. *Commun. Biol.* **6**, 578 (2023). 37253813 10.1038/s42003-023-04817-y PMC10229695

[9] Minns, M. S. et al. NLRP3 selectively drives IL-1β secretion by *Pseudomonas aeruginosa* infected neutrophils and regulates corneal disease severity. *Nat. Commun.* **14**, 5832 (2023). 37730693 10.1038/s41467-023-41391-7 PMC10511713

[10] Zhong, Z. et al. New mitochondrial DNA synthesis enables NLRP3 inflammasome activation. *Nature* **560**, 198–203 (2018). 30046112 10.1038/s41586-018-0372-z PMC6329306

[11] Subramanian, N., Natarajan, K., Clatworthy, M. R., Wang, Z. & Germain, R. N. The adaptor MAVS promotes NLRP3 mitochondrial localization and inflammasome activation. *Cell* **153**, 348–361 (2013). 23582325 10.1016/j.cell.2013.02.054 PMC3632354

[12] Wang, B., Han, J., Elisseeff, J. H. & Demaria, M. The senescence-associated secretory phenotype and its physiological and pathological implications. *Nat. Rev. Mol. Cell Biol.* **25**, 958–978 (2024). 38654098 10.1038/s41580-024-00727-x

[13] McArthur, K. et al. BAK/BAX macropores facilitate mitochondrial herniation and mtDNA efflux during apoptosis. *Science*10.1126/science.aao6047 (2018). 10.1126/science.aao6047 29472455

[14] Kinoshita, T., Wang, Y., Hasegawa, M., Imamura, R. & Suda, T. PYPAF3, a PYRIN-containing APAF-1-like protein, is a feedback regulator of caspase-1-dependent interleukin-1beta secretion. *J. Biol. Chem.* **280**, 21720–21725 (2005). 15817483 10.1074/jbc.M410057200

[15] Mukherjee, S. P., Quintas, P. O., McNulty, R., Komives, E. A. & Dyson, H. J. Structural characterization of the ternary complex that mediates termination of NF-kappaB signaling by IkappaBalpha. *Proc. Natl. Acad. Sci. USA* **113**, 6212–6217 (2016). 27185953 10.1073/pnas.1603488113 PMC4896678

[16] Vacca, M. et al. NLRP10 enhances CD4(+) T-cell-mediated IFNgamma response via regulation of dendritic cell-derived IL-12 release. *Front. Immunol.* **8**, 1462 (2017). 29163529 10.3389/fimmu.2017.01462 PMC5673625

[17] Yi, X. et al. NLRP10 promotes AGEs-induced NLRP1 and NLRP3 inflammasome activation via ROS/MAPK/NF-kappaB signaling in human periodontal ligament cells. *Odontology* **112**, 100–111 (2024). 37043073 10.1007/s10266-023-00813-0

[18] Prochnicki, T. et al. Mitochondrial damage activates the NLRP10 inflammasome. *Nat. Immunol.* **24**, 595–603 (2023). 36941400 10.1038/s41590-023-01451-y

[19] Radom, C. T., Banerjee, A. & Verdine, G. L. Structural characterization of human 8-oxoguanine DNA glycosylase variants bearing active site mutations. *J. Biol. Chem.* **282**, 9182–9194 (2007). 17114185 10.1074/jbc.M608989200

[20] Hornung, V. et al. Silica crystals and aluminum salts activate the NALP3 inflammasome through phagosomal destabilization. *Nat. Immunol.* **9**, 847–856 (2008). 18604214 10.1038/ni.1631 PMC2834784

[21] Elliott, E. I. et al. Cutting edge: mitochondrial assembly of the NLRP3 inflammasome complex is initiated at priming. *J. Immunol.* **200**, 3047–3052 (2018). 29602772 10.4049/jimmunol.1701723 PMC5916517

[22] Mestas, J. & Hughes, C. C. Of mice and not men: differences between mouse and human immunology. *J. Immunol.* **172**, 2731–2738 (2004). 14978070 10.4049/jimmunol.172.5.2731

[23] Volchuk, A., Ye, A., Chi, L., Steinberg, B. E. & Goldenberg, N. M. Indirect regulation of HMGB1 release by gasdermin D. *Nat. Commun.* **11**, 4561 (2020). 32917873 10.1038/s41467-020-18443-3 PMC7486936

[24] Schatz, G. & Dobberstein, B. Common principles of protein translocation across membranes. *Science* **271**, 1519–1526 (1996). 8599107 10.1126/science.271.5255.1519

[25] Meng, E. C. et al. UCSF ChimeraX: tools for structure building and analysis. *Protein Sci.* **32**, e4792 (2023). 37774136 10.1002/pro.4792 PMC10588335

[26] Almagro Armenteros, J. J., Sonderby, C. K., Sonderby, S. K., Nielsen, H. & Winther, O. DeepLoc: prediction of protein subcellular localization using deep learning. *Bioinformatics* **33**, 3387–3395 (2017). 29036616 10.1093/bioinformatics/btx431

[27] Cabral, J. E. et al. Targeting the NLRP3 inflammasome for inflammatory disease therapy. *Trends Pharmacol. Sci*. 10.1016/j.tips.2025.04.007 (2025). 10.1016/j.tips.2025.04.007 PMC12570976 40374417

[28] de Almeida, L. et al. The PYRIN domain-only protein POP1 inhibits inflammasome assembly and ameliorates inflammatory disease. *Immunity* **43**, 264–276 (2015). 26275995 10.1016/j.immuni.2015.07.018 PMC4666005

[29] Gangopadhyay, A. et al. NLRP3 licenses NLRP11 for inflammasome activation in human macrophages. *Nat. Immunol.* **23**, 892–903 (2022). 35624206 10.1038/s41590-022-01220-3 PMC9174058

[30] You, Q., Feng, X., Cai, Y., Baylin, S. B. & Li, H. Human 8-oxoguanine glycosylase OGG1 binds nucleosome at the dsDNA ends and the super-helical locations. *Commun. Biol.* **7**, 1202 (2024). 39341999 10.1038/s42003-024-06919-7 PMC11438860

[31] Bruner, S. D., Norman, D. P. & Verdine, G. L. Structural basis for recognition and repair of the endogenous mutagen 8-oxoguanine in DNA. *Nature* **403**, 859–866 (2000). 10706276 10.1038/35002510

[32] Hernandez-Segura, A., Nehme, J. & Demaria, M. Hallmarks of cellular senescence. *Trends Cell Biol.* **28**, 436–453 (2018). 29477613 10.1016/j.tcb.2018.02.001

[33] Baker, D. J. et al. Naturally occurring p16(Ink4a)-positive cells shorten healthy lifespan. *Nature* **530**, 184–189 (2016). 26840489 10.1038/nature16932 PMC4845101

[34] Dou, Z. et al. Cytoplasmic chromatin triggers inflammation in senescence and cancer. *Nature* **550**, 402–406 (2017). 28976970 10.1038/nature24050 PMC5850938

[35] Li, C. et al. DNA damage-triggered activation of cGAS-STING pathway induces apoptosis in human keratinocyte HaCaT cells. *Mol. Immunol.* **131**, 180–190 (2021). 33423764 10.1016/j.molimm.2020.12.037

[36] Gov, L., Schneider, C. A., Lima, T. S., Pandori, W. & Lodoen, M. B. NLRP3 and potassium efflux drive rapid IL-1beta release from primary human monocytes during *Toxoplasma gondii* infection. *J. Immunol.* **199**, 2855–2864 (2017). 28904126 10.4049/jimmunol.1700245 PMC5648586

[37] Todoric, J. et al. Fructose stimulated de novo lipogenesis is promoted by inflammation. *Nat. Metab.* **2**, 1034–1045 (2020). 32839596 10.1038/s42255-020-0261-2 PMC8018782

[38] Lackner, A., Leonidas, L., Macapagal, A., Lee, H. & McNulty, R. How interactions between oxidized DNA and the NLRP3 inflammasome fuel inflammatory disease. *Trends Biochem. Sci.* **50**, 931–944 (2025). 40883132 10.1016/j.tibs.2025.07.007 PMC12564193

[39] Hochheiser, I. V. et al. Structure of the NLRP3 decamer bound to the cytokine release inhibitor CRID3. *Nature* **604**, 184–189 (2022). 35114687 10.1038/s41586-022-04467-w

[40] Lackner, A. et al. Measuring interactions between proteins and small molecules or nucleic acids. *Curr Protoc.* **4**, e1105 (2024). 10.1002/cpz1.1105 PMC11335060 39040024

[41] Cabral. A., Cabral, J. E. & McNulty, R. Cryo-EM for small molecules. *Curr Protoc.* **2**, e632 (2022). 10.1002/cpz1.632 PMC11245322 36511652

