# Supplementary material for: NLRP10 engages oxidized DNA through a Schiff-base mechanism and dissociates from NLRP3 upon inflammasome activation
Source: Commun Biol. 2026 Jan 22;9:72. doi: 10.1038/s42003-025-09501-x (PMC12827243; doi:10.1038/s42003-025-09501-x)
Supplement: Supplementary file 1 — Supplementary Information [file 42003_2025_9501_MOESM1_ESM.pdf]

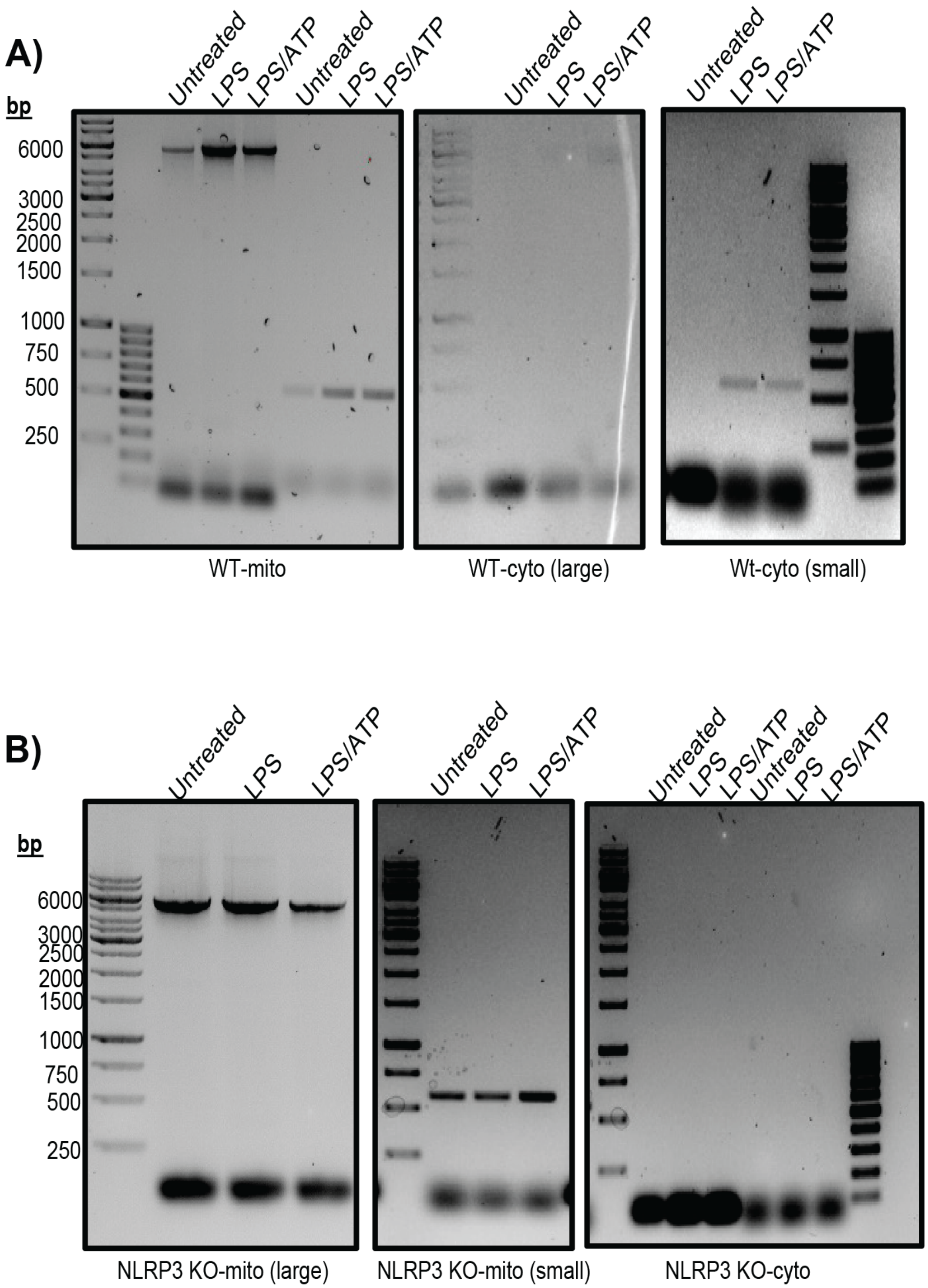

Supplementary Figure 1. Uncropped DNA gels show mtDNA release dependent on NLRP3 (Fig. 1A Uncropped). A) Uncropped agarose gels for amplified small and large fragments in WT iBMDM's. **B)** Uncropped agarose gels for amplified small and large fragments in NLRP3 KO iBMDM's.

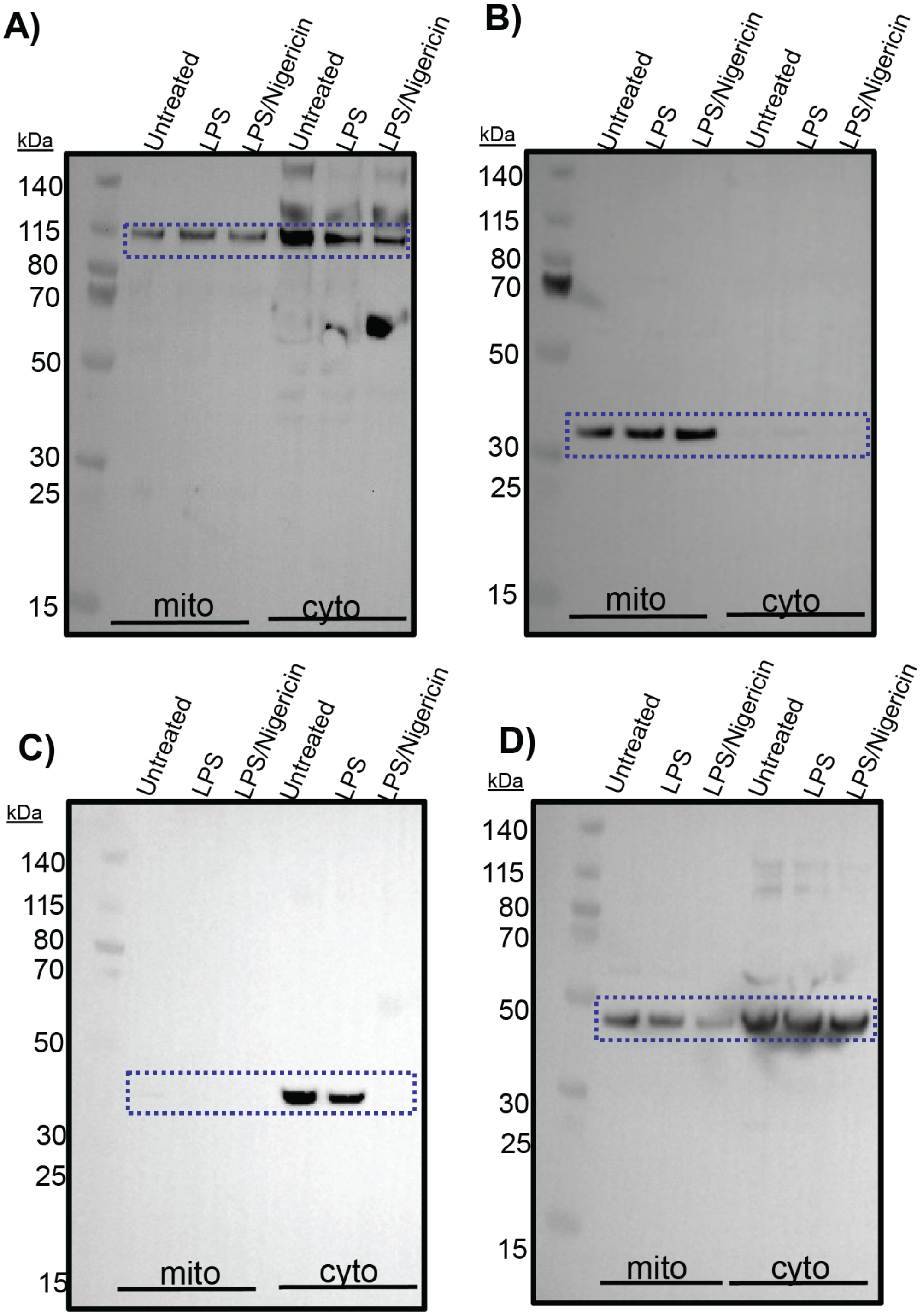

Supplementary Figure 2. Uncropped westerns show NLRP3 mitochondrial association (Fig. 1B Uncropped). A) Uncropped representative anti-NLRP3 western blot. **B)** Uncropped representative anti-VDAC western blot. **C)** Uncropped representative anti-GAPDH western blot. **D)** Uncropped representative anti-β-actin western blot.

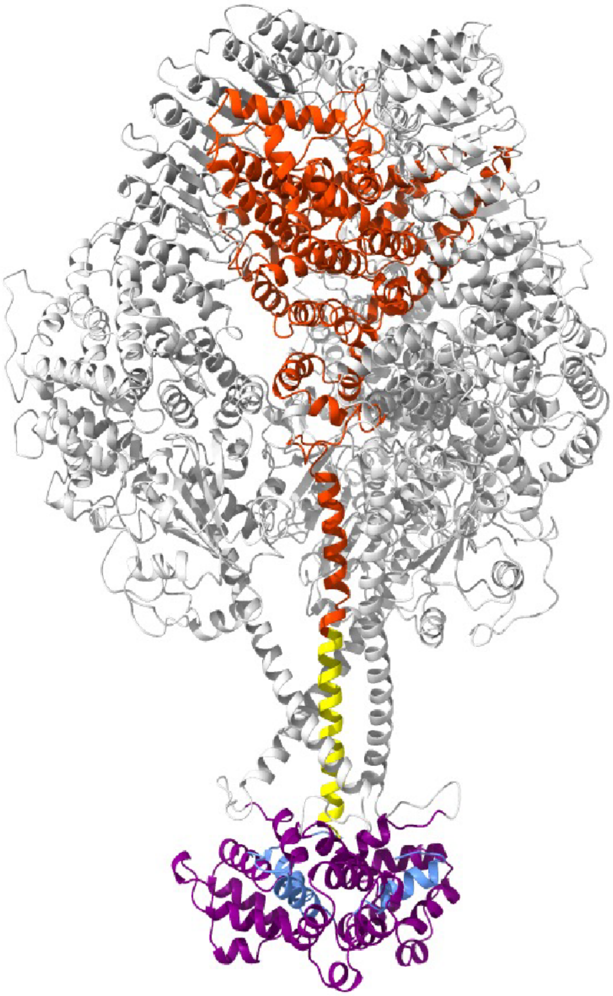

MAVS: 1-513
MAVS Transmembrane Domain: 514-540
NLRP3 Monomers: 94-1036
NLRP3 Pyrin Domain: 1-93
NLRP3 N-terminal Helix: 1-17

Supplementary Figure 3. NLRP3 predicted to interact with MAVS. AlphaFold3 model of 3 NLRP3 monomers and MAVS. MAVS (1-513); MAVS Transmembrane Domain (514-540); NLRP3 Monomers (94-1036); NLRP3 Pyrin Domain (1-93); NLRP3 N-terminal helix (1-17).

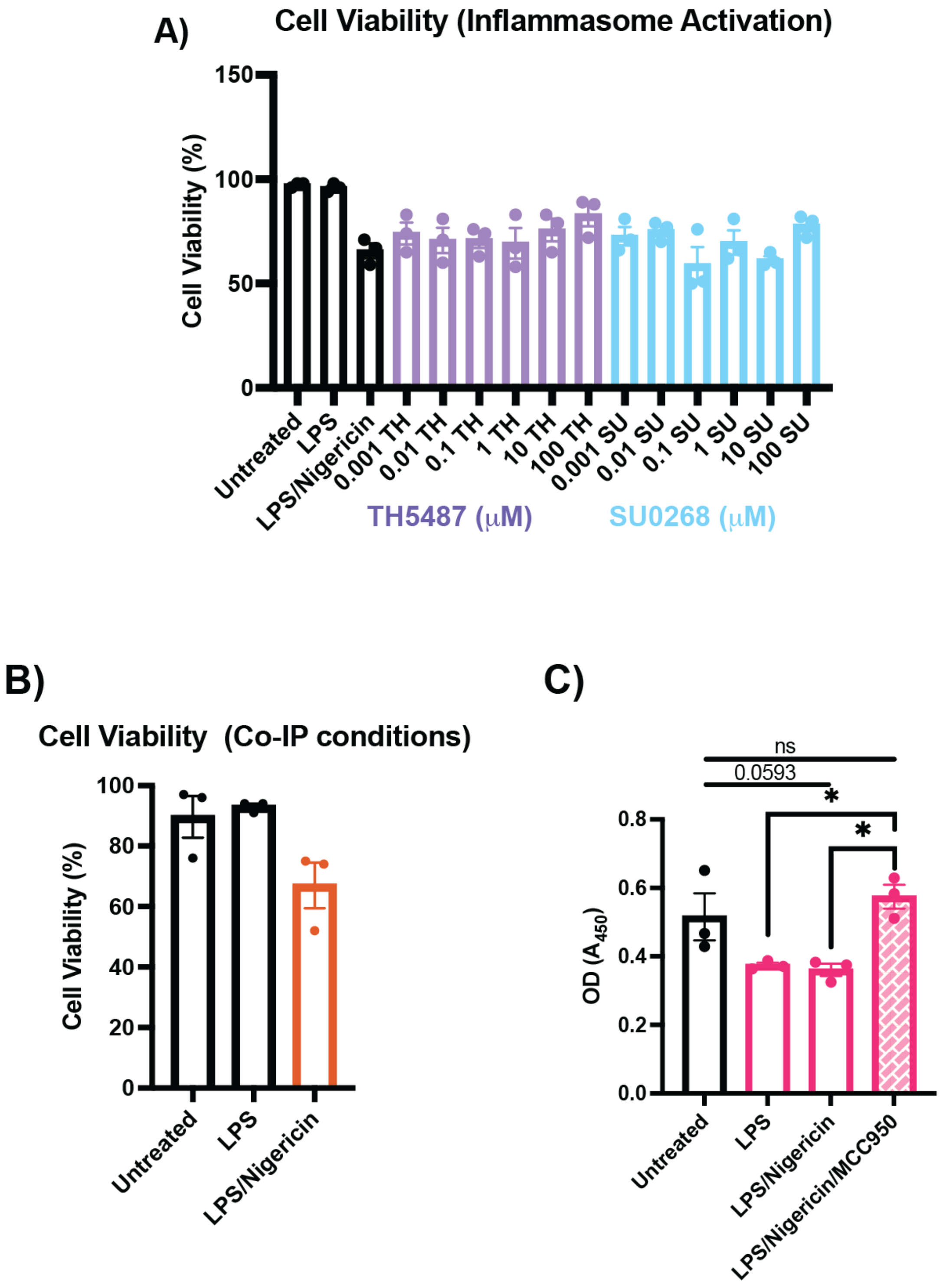

Supplementary Figure 4. THP-1 Cell viability and NLRP10/NLRP3 association. A) Trypan blue staining of THP-1 cells following treatment with or without TH5487 or SU0268. Data points represent individual biological replicates. N=3. **B)** Trypan blue staining of THP-1 cells following treatment with or without LPS/nigericin for co-immunoprecipitation. Data points represent individual biological replicates. N=3. **C)** ELISA performed to assess the interaction between NLRP10 and NLRP3 under inflammasome activation treated with or without MCC950 (10 μM). Error bars: mean ± SEM, analyzed with one-way ANOVA. N=3, ∗p=0.0130, 0.0182.

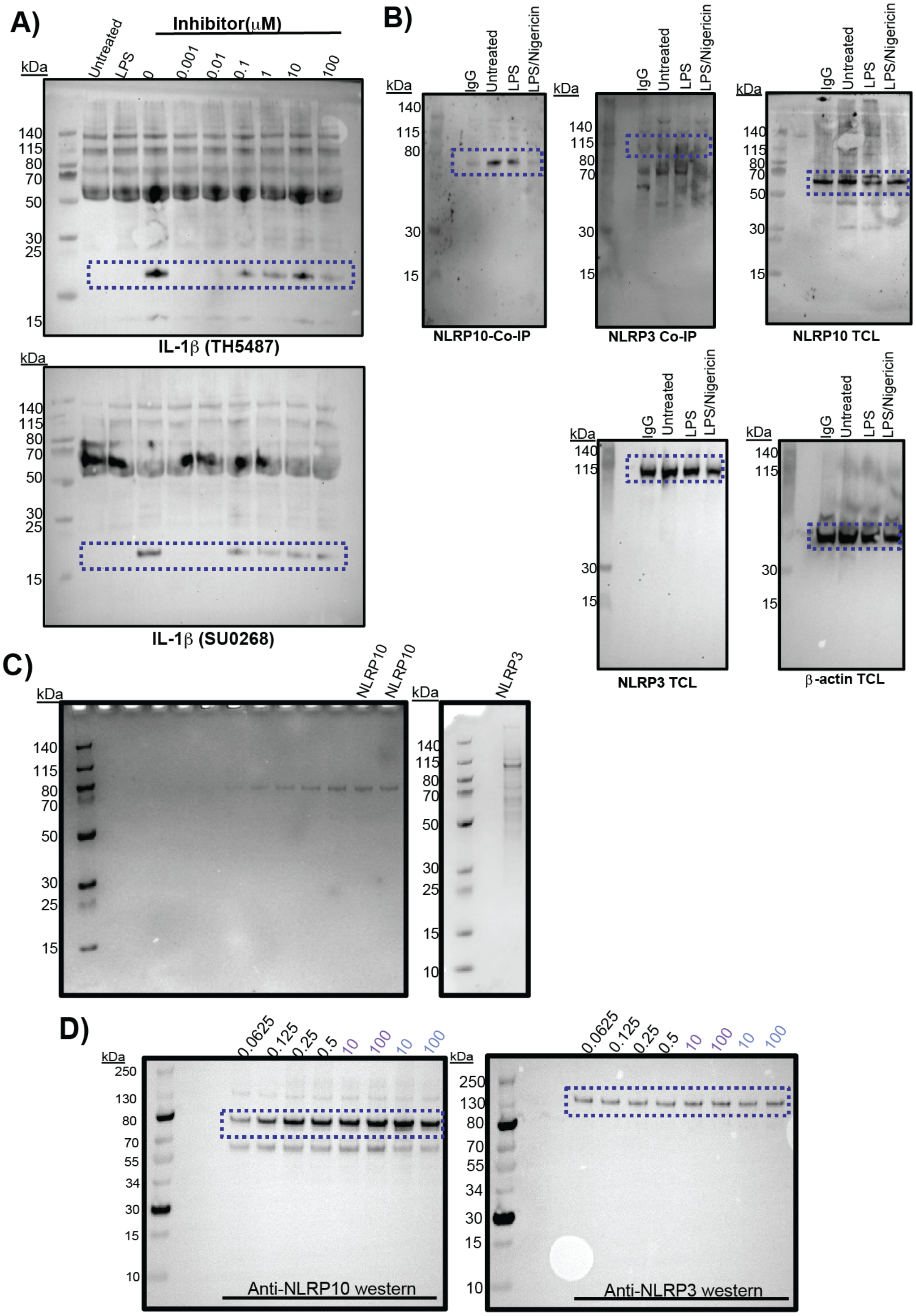

Supplementary Figure 5. Uncropped westerns. A) Uncropped western blots showing representative blots of IL-1β secretion in THP-1 cells activated with nigericin and treated with TH5487 (top - Fig. 3A) or SU0268 (bottom - Fig. 3B). **B)** Uncropped western blots showing representative blots of co-immunoprecipitation assays (Fig. 3C). **C)** Uncropped western blots showing NLRP10 and NLRP3 purification (Fig. 3E). **D)** Uncropped western blots showing co-immunoprecipitation assays with purified protein (Fig. 3F).

A)

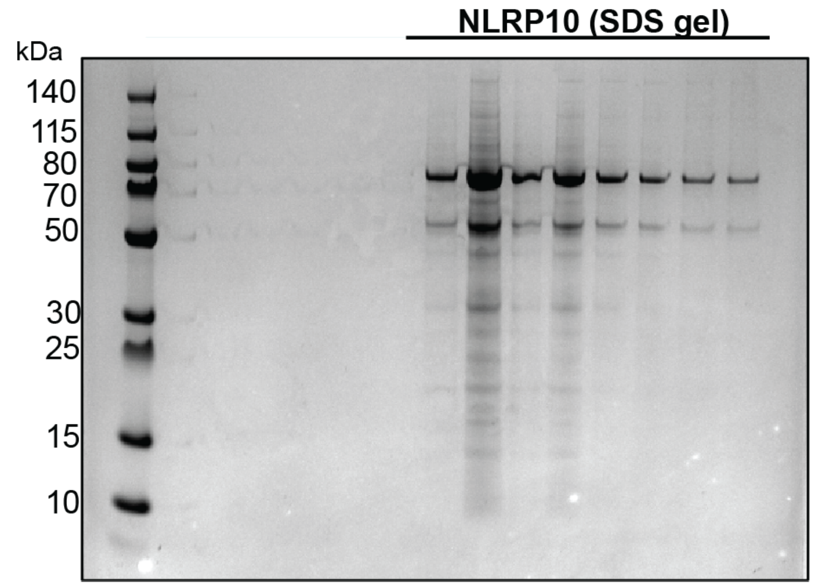

B)

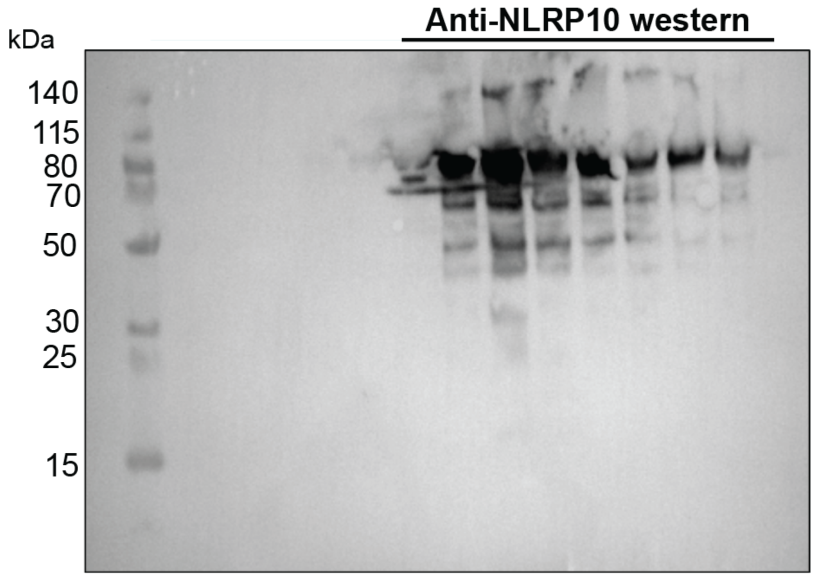

Supplementary Figure 6. Uncropped blots for NLRP10 purification (Fig. 4C Uncropped). A) SDS gel following FLAG column. **B)** Anti-NLRP10 western performed following FLAG column.

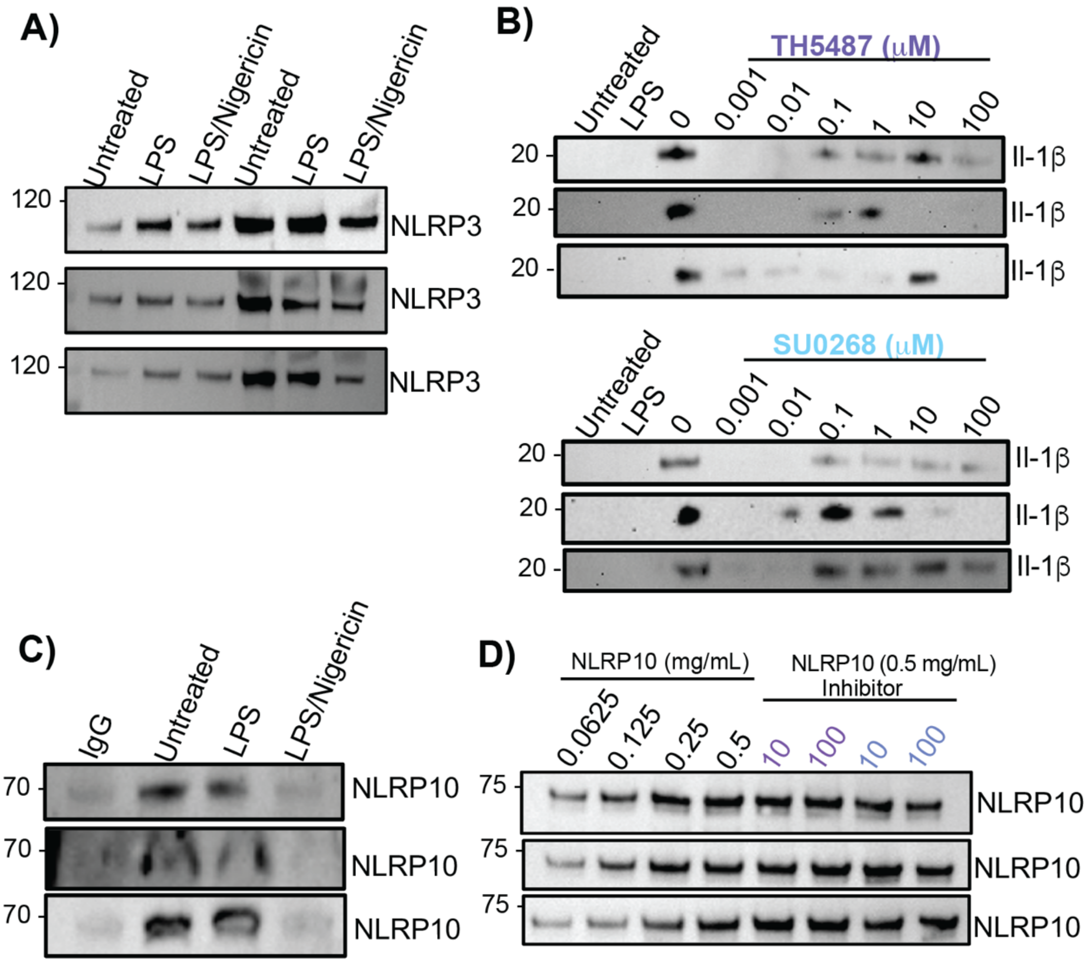

Supplementary Figure 7. Blots used for quantification. A) Anti-NLRP3 western blots showing mitochondrial association. **B)** Anti-IL-1β western blots show decreased secretion with treatment with TH5487 or SU0268. **C)** Anti-NLRP10 western blots show decreased association with NLRP3 under activating conditions. **D)** Anti-NLRP10 western blots show NLRP10 and NLRP3 association with purified protein.
