# Supplementary material for: NLRP10 engages oxidized DNA through a Schiff-base mechanism and dissociates from NLRP3 upon inflammasome activation
Source: Commun Biol. 2026 Jan 22;9:72. doi: 10.1038/s42003-025-09501-x (PMC12827243; doi:10.1038/s42003-025-09501-x)
Supplement: Supplementary file 2 — Reporting Summary [file 42003_2025_9501_MOESM2_ESM.pdf]

nature portfolio

Corresponding author(s): Reginald McNulty

Last updated by author(s): Dec 12, 2025

Reporting Summary

Nature Portfolio wishes to improve the reproducibility of the work that we publish. This form provides structure for consistency and transparency in reporting. For further information on Nature Portfolio policies, see our Editorial Policies and the Editorial Policy Checklist.

Statistics

For all statistical analyses, confirm that the following items are present in the figure legend, table legend, main text, or Methods section.

n/a	Confirmed	
☐	☒	The exact sample size (*n*) for each experimental group/condition, given as a discrete number and unit of measurement
☐	☒	A statement on whether measurements were taken from distinct samples or whether the same sample was measured repeatedly
☐	☒	The statistical test(s) used AND whether they are one- or two-sided<br>*Only common tests should be described solely by name; describe more complex techniques in the Methods section.*
☒	☐	A description of all covariates tested
☒	☐	A description of any assumptions or corrections, such as tests of normality and adjustment for multiple comparisons
☐	☒	A full description of the statistical parameters including central tendency (e.g. means) or other basic estimates (e.g. regression coefficient) AND variation (e.g. standard deviation) or associated estimates of uncertainty (e.g. confidence intervals)
☒	☐	For null hypothesis testing, the test statistic (e.g. *F*, *t*, *r*) with confidence intervals, effect sizes, degrees of freedom and *P* value noted<br>*Give P values as exact values whenever suitable.*
☒	☐	For Bayesian analysis, information on the choice of priors and Markov chain Monte Carlo settings
☒	☐	For hierarchical and complex designs, identification of the appropriate level for tests and full reporting of outcomes
☒	☐	Estimates of effect sizes (e.g. Cohen's *d*, Pearson's *r*), indicating how they were calculated

Our web collection on statistics for biologists contains articles on many of the points above.

Software and code

Policy information about availability of computer code

Data collection	NA
Data analysis	Prism and ChimeraX

For manuscripts utilizing custom algorithms or software that are central to the research but not yet described in published literature, software must be made available to editors and reviewers. We strongly encourage code deposition in a community repository (e.g. GitHub). See the Nature Portfolio guidelines for submitting code & software for further information.

Data

Policy information about availability of data

All manuscripts must include a data availability statement. This statement should provide the following information, where applicable:

- Accession codes, unique identifiers, or web links for publicly available datasets
- A description of any restrictions on data availability
- For clinical datasets or third party data, please ensure that the statement adheres to our policy

Data analysis was performed in Prism, and the results are available at: https://figshare.com/s/cb80e47bc3bd0159cf54. Additional data supporting this study are available from the corresponding author upon reasonable request.

Research involving human participants, their data, or biological material

Policy information about studies with human participants or human data. See also policy information about sex, gender (identity/presentation), and sexual orientation and race, ethnicity and racism.

Reporting on sex and gender	NA
Reporting on race, ethnicity, or other socially relevant groupings	NA
Population characteristics	NA
Recruitment	NA
Ethics oversight	NA

Note that full information on the approval of the study protocol must also be provided in the manuscript.

Field-specific reporting

Please select the one below that is the best fit for your research. If you are not sure, read the appropriate sections before making your selection.

☒ Life sciences ☐ Behavioural & social sciences ☐ Ecological, evolutionary & environmental sciences

For a reference copy of the document with all sections, see nature.com/documents/nr-reporting-summary-flat.pdf

Life sciences study design

All studies must disclose on these points even when the disclosure is negative.

Sample size	NA
Data exclusions	NA
Replication	All experiments were peformed with three biological replicates
Randomization	NA
Blinding	NA

Reporting for specific materials, systems and methods

We require information from authors about some types of materials, experimental systems and methods used in many studies. Here, indicate whether each material, system or method listed is relevant to your study. If you are not sure if a list item applies to your research, read the appropriate section before selecting a response.

Materials & experimental systems

n/a	Involved in the study	
☐	☒	Antibodies
☐	☒	Eukaryotic cell lines
☒	☐	Palaeontology and archaeology
☒	☐	Animals and other organisms
☒	☐	Clinical data
☒	☐	Dual use research of concern
☒	☐	Plants

Methods

n/a	Involved in the study	
☒	☐	ChIP-seq
☒	☐	Flow cytometry
☒	☐	MRI-based neuroimaging

Antibodies

Antibodies used	NLRP3 Pyrin targeting (Adipogen–AG-20B-0014-C100), VDAC (Cell Signaling - #4661), b-actin (Santa Cruz - #47778), NLRP10 (Cell signaling -E5X5A), NLRP10 (R&D systems MAB6606), GAPDH Antibody (Santa Cruz 47724), 8-oxo-dG antibody (Rockland 200-301-A99), Alexa Fluor 488 goat anti-mouse IgG/IgM (H+L) (Thermo Fisher), Anti-mouse IgG, HRP-linked Antibody (Cell Signaling 7076S), Anti-rabbit HPR antibody (Cell Signaling 7074S), NLRP3 polyclonal antibody (ABclonal A12694).

Validation	Adipogen–AG-20B-0014-C100: Published previously in Commun Biol. PMID 37253813<br>VDAC (Cell Signaling - #4661): Human Reactive<br>β-actin (Santa Cruz - #47778): detection of β-Actin of mouse, rat, human<br>NLRP10 (Cell signaling -E5X5A): human reactive<br>NLRP10 (R&D systems MAB6606): Detects human NLRP10/Pynod/NALP10 in direct ELISAs and Western blots. In direct ELISAs, no cross-reactivity with recombinant human (rh) NALP1 (aa 1 323), rhNALP1 (aa 1331 1429), rhNALP2 (aa 1 208), rhNALP3 (aa 3 223), rhNALP3 (aa 540 689), rhNALP5 (aa 469 635), rhNOD1 (aa 830 953), or rhNOD2 (aa 230 450) is observed.<br>GAPDH Antibody (Santa Cruz 47724):GAPDH of human origin by WB, IP, IF and IHC(P)<br>8-oxo-dG antibody (Rockland 200-301-A99): This Protein G purified antibody has been tested for use in immunohistochemistry, ICC/IF, Dot Blot, IP, Flow Cytometry, and ELISA<br>NLRP3 polyclonal antibody (ABclonal A12694): WB (Western Blot)Human, Mouse, Rat, Chicken, Other

Eukaryotic cell lines

Policy information about cell lines and Sex and Gender in Research

Cell line source(s)	iBMDM (WT and NLRP3 KO), THP-1, Expi293 expression cells
Authentication	THP1 cell were purchased from Invivogen and iBMDM were provided from Micheal Karin
Mycoplasma contamination	Cell lines were confirmed negative for mycoplasma
Commonly misidentified lines<br>(See ICLAC register)	NA

Plants

Seed stocks	NA
Novel plant genotypes	NA
Authentication	NA
